# Clinic-Based Infant Screening for Duchenne Muscular Dystrophy: A Feasibility Study

**DOI:** 10.1371/4f99c5654147a

**Published:** 2012-05-02

**Authors:** Alissa Cyrus, Natalie Street, Sharon Quary, Julie Kable, Aileen Kenneson, Paul Fernhoff

## Abstract

Purpose. The purpose of this study was to assess the desirability of Duchenne muscular dystrophy (DMD) screening, the effectiveness of the consent process, and the feasibility of conducting DMD screening in a pediatric office. Methods. Infant males who attended a 12-month routine well-child visit at a participating pediatric clinic were screened for DMD. Parents and providers completed post-screening questionnaires to assess their experiences with and attitudes toward screening. Results. A total of 264 male infants were screened for DMD. Approximately 78% of parents indicated support of voluntary DMD screening and 91% of providers were in favor of screening for DMD. About 75% of parents correctly answered three of five questions testing their knowledge of DMD screening. Conclusion. DMD screening is feasible in a pediatric office when conducted as part of a research study. Infant screening for DMD eventually could be offered in pediatric health care provider offices as an optional public health service outside of newborn screening.

## Introduction

Duchenne muscular dystrophy (DMD) is the most common childhood muscular dystrophy (OMIM #310200). Birth prevalence of DMD ranges from about 1 in 3,900 to 1 in 6,000 live male births1, 2. DMD is an X-linked condition that affects males primarily and might or might not occur in the presence of a family history^3^. Signs of DMD often appear before a child is 3 years of age and include gross motor delay, muscle weakness, and difficulty walking and running^4^. Delays in diagnosis of DMD are well-documented4, 5, 6, 7. A delay of more than 2 years between symptom onset and diagnosis is common because early signs are variable and nonspecific to DMD^4^. Despite advances in treatment and diagnostic testing, the average age of diagnosis is 5 years and has not changed in the past two decades^4^.

Obtaining an earlier diagnosis of DMD has several benefits to an affected child and his family. Some of these benefits are: (1) decreasing the cost and stress associated with numerous visits to health care providers in establishing a diagnosis (known as the “diagnostic odyssey”); (2) enabling affected children to have earlier access to supportive therapies and services; (3) allowing parents to prepare for having a child with a disability; and (4) providing reproductive knowledge to parents and other family members soon after the birth of an affected child1, 8, 9. In the 1970s, early diagnosis became possible through detection of elevated creatine kinase (CK) in dried blood spots^10^. Since then, several DMD screening pilot studies and programs have occurred worldwide1, 2, 8, 10, 11, 12, 13, 14, 15, 16. Currently, DMD newborn screening is only being conducted in Antwerp (François Eyskens, personal communication, November 7, 2011).

Although newborn screening for DMD has clear benefits, it has not been adopted more widely because DMD does not meet certain criteria used to determine which conditions become part of traditional newborn screening programs^17^. In particular, DMD does not have an accepted medical treatment that prevents or reverses disease manifestations. Although experts recommend supportive therapies for DMD that slow progression and increase well-being, little evidence is available to suggest earlier access to these therapies―particularly those started in the newborn period―improves clinical outcomes18, 19, 20, 21, 22. Given the lack of early, definitive treatment, some parents might not want to know if their newborn has DMD. As a result, DMD newborn screening programs typically have been voluntary and have focused primarily on benefits of early diagnosis to the family. Even so, ethicists have asked whether parents can be informed adequately about the risks and benefits of screening for DMD when receiving information during the busy newborn period^23^. Additional concerns have been raised about the potential for disrupting parent–newborn bonding upon receiving a presymptomatic diagnosis of a fatal disorder and the burden for parents receiving false-positive results24, 25.

Offering DMD screening after the newborn period―but before the onset of symptoms―might address many of the ethical concerns raised about newborn screening, while also realizing the benefits of early detection^23^. Information could be provided to parents after they have had time to bond with their infant and to consider the potential consequences of receiving positive screening results. Screening could be offered at the age when affected boys would still be presymptomatic and most parents would not have had additional children^26^.

In 2004, the Centers for Disease Control and Prevention (CDC) convened a workshop to discuss past and present DMD newborn screening programs, the risks and benefits of screening, and to announce the availability of funding for newborn and older infant DMD screening studies (http://www.cdc.gov/ncbddd/duchenne/documents/NBS_Lay_Report.pdf). As a result, two DMD screening studies were conducted: (1) a pilot newborn screening study in Ohio^27^,and (2) a pilot infant screening study in metropolitan Atlanta. Here we report results from the infant DMD screening study, in which parents were offered DMD screening for their infant sons at the 12-month pediatric clinic visit. The goals of this study were to determine: (1) parental experience with and desirability of DMD screening; (2) how well the consent process informed parents; and (3) the logistical feasibility of conducting DMD screening in a pediatric office setting.

## Materials and Methods

Study recruitment took place at four metropolitan Atlanta pediatric clinics from February through September 2007. Male infants who attended the 12-month routine well-child visit at one of the participating pediatric clinics were eligible for the study. To avoid the increased risk for false-positive screens, infants were excluded if they were seeing a pediatrician because of illness. Infants with a family history of DMD also were excluded, as these infants and their families would have required a more extensive genetic evaluation.

Parents were given a study packet to review that contained an introductory letter, brochure, consent form, Health Insurance Portability and Accountability Act form, and decliner questionnaire. Study staff then discussed the study materials with all parents during the visit, answered questions, and obtained consent. Parents were given the option to have the sample held for 2 weeks while they took additional time to review the study packet. After 2 weeks, study staff called the parent(s) for a final decision about participation. A toll-free number and a website were available to parents wanting more information about the study. Parents also were given the option of withdrawing from the study at any time.

The screening test used for this study detected total CK in dried blood spots on filter paper^28^. Sample collection for DMD screening was performed at the 12-month visit. As the American Academy of Pediatrics recommends testing infants 12 months of age for anemia^29^, this visit was ideal for DMD screening because a sample could be obtained using blood from the same fingerstick. Office staff involvement was limited to performing the fingerstick. Two extra drops of blood were collected on a separate filter paper card to be used for DMD screening. Following blood collection, the card was transported by study staff to the Emory Biochemical Genetics Laboratory where the CK testing was performed.

Negative screening test results were mailed to parents approximately 1 to 2 weeks after sample collection. Included in the results packet were a result letter; a post-screening questionnaire; and a preaddressed, postage-paid envelope. Parents were asked to complete the questionnaire and return it in the envelope provided. They also were given the option to complete a questionnaire online. Follow-up telephone calls were made to ensure receipt of study results and to remind parents to complete the questionnaire. A second questionnaire was mailed approximately a month after the results were sent. Parents were asked to complete and return the questionnaires only if they had not previously done so. Parents of twins were instructed to complete only one questionnaire. A protocol to follow-up infants with elevated CK (positive screening result) was also developed.

Self-Administered Post-Screening Questionnaires

Investigators from the newborn and infant screening pilot studies developed questionnaires that assessed the experiences of parents and health care providers. The post-screening questionnaire given to parents whose sons participated in the screening program evaluated: (1) overall experience with the screening program; (2) the value of the written and verbal study information parents received; (3) basic knowledge about DMD and screening; and (4) demographic and family history information. The decliner questionnaire evaluated factors that influenced refusal of screening for parents who received information about the study, but declined participation. This questionnaire was included in the study packet. Parents could complete and return it during the appointment or in a preaddressed stamped envelope. The provider questionnaire was designed for providers in participating clinics to assess their attitudes and opinions about DMD screening and its incorporation into their clinical practice. Pediatricians, physician assistants, nurses, nurse practitioners, and medical assistants involved in sample collection were asked to fill out the questionnaire after completion of the study.

Statistical Analysis

Questionnaire data were encoded using SurveyMonkey™ (www.surveymonkey.com, LLC, Palo Alto, CA). Data were verified and then imported into Statistical Package for the Social Sciences (SPSS) version 15.0. for analysis^30^. Descriptive statistics were computed to characterize the responses. To assess differences between groups,* t* tests for continuous data and chi-squares for categorical data were computed. Statistical significance was determined to be *P* < 0.05.

Other Considerations

An advisory group composed of individuals with varying expertise in DMD (i.e. health care providers, Muscular Dystrophy Association representatives, and parents of males with DMD) was formed to provide guidance and oversight for the infant screening study. The institutional review boards (IRBs) of Emory University and CDC approved the study protocol.

## Results

From February 1, 2007 through September 30, 2007, the four clinics scheduled a total of 524 infant males for the 12-month well-baby visit. Study staff approached 352 parents of 361 of these infants about participation in the study. A total of 266 (75.6%) parents of 273 infant males (including 7 pairs of twins) consented to DMD screening, 69 (19.6%) parents of 71 infant males (including 2 pairs of twins) declined, and 17 (4.8%) infant males were excluded.

Although parents of 273 infants had consented to screening, blood samples were collected from only 264 males. The nine infants without samples either had left prior to receiving the fingerstick or already had the fingerstick for the anemia test at a previous visit and no extra blood had been collected for DMD screening. The samples were analyzed for total CK. The mean age at sample collection was 12.31 months (range: 11–15 months; standard deviation [SD] = 0.6). The mean CK level detected was 113.8 units/liter (U/L) (range: 12–221 U/L; SD = 37.1). All samples fell within the normal CK reference range. Thus, no further testing was performed and all infant males received a negative screening result.

Demographics of Questionnaire Respondents

Of the 259 parents of the 264 boys (5 pairs of twins) screened, 138 (53.3%) returned completed post-screening questionnaires, about half from the first mailing and half from the second mailing. Demographics of questionnaire respondents are described in **Table 1**. Mothers were the primary respondents to the questionnaire. Most respondents (91.9%) were married. The respondents were well educated; 35.1% had completed a 4-year college degree and 55.2% had completed post-graduate study. Approximately 80% of the sample described themselves as White, and 13% as Black or African American. Only 3.7% of the sample indicated that they were Hispanic or Latino.

**Table 1. Demographic characteristics of parents who accepted or declined Duchenne muscular dystrophy screening for their sons and of providers who completed a questionnaire:** 

Characteristic	Participant (n = 138*)	Decliner (n = 22)*	Provider (n = 25)*
*Race, n (%)* Black or African American White Other	18 (13.2) 108 (79.4) 10 (7.4)	3 (13.6) 16 (72.7) 3 (13.6)	4 (18.2) 17 (77.3) 1 (4.5)
*Ethnicity, n (%)* Hispanic or Latino Non-Hispanic or non-Latino	5 (3.7) 131 (96.3)	0 (0) 22 (100)	0 (0) 22 (100)
*Relationship to child, n (%)* Mother Father Other	120 (88.2) 15 (11.0) 1 (1.7)	20 (90.9) 2 (9.1) 0 (0)	
*Marital Status, n (%)* Married Not married	125 (91.9) 11 (8.0)	18 (94.7) 1 (5.3)	
*Education, n (%)* 4-year college graduate Post-graduate study Other	47 (35.1) 74 (55.2) 13 (9.7)	10 (52.6) 9 (47.4) 0 (0)	
*Sex, n (%)* Male Female			5 (22.7) 17 (77.3)
*Role, n (%)* Physician Nurse Other			7 (31.8) 9 (40.9) 6 (27.2)
*Years in practice, mean (range)*			15.7 (2-37)
*Total n varies for each characteristic due to missing responses.**			

Twenty-two (31.0%) of the sixty-nine parents who declined participation returned decliner questionnaires (**Table 1**). The respondents who declined were similar to those who participated. Mothers were the parents who usually completed the questionnaire and most indicated they were married. Approximately 78% of the sampled parents identified themselves as White and 13% as Black or African American. None of the respondents identified themselves as being Hispanic or Latino. All respondents indicated that they had completed either a 4-year college degree or post-graduate study. No statistical differences in the demographics of the participants and decliners were found.

Reasons for Participating or Declining Screening

Parents whose sons participated in screening and those whose sons did not were asked their three most important reasons for participating in or declining screening. Responses that were reported by a minimum of 10% of participants and decliners are summarized in **Table 2**. The three most common reasons for accepting screening were: (1) “I just wanted to know”; (2) “If he had DMD, I wanted to get treatment for my son earlier”; and (3) “I wanted to rule it out to have one less thing to worry about”. In contrast, the three most frequently reported reasons for declining screening were: (1) “My child is healthy”; (2) “No other family member has DMD”; and (3) “There is no real benefit of knowing right now”.

**Table 2. Parents’ most frequently reported reasons for accepting or declining Duchenne muscular dystrophy screening for their sons:** 

Participants (n = 138)	%	Decliners (n = 22)	%
I just wanted to know.	54.3	My child is healthy	63.6
If he had DMD, I wanted to get treatment for my son earlier.	42.0	No other family member with DMD.	45.5
I wanted to rule it out to have one less thing to worry about.	31.2	There is no real benefit of knowing right now.	22.7
If he had DMD, I wanted to be able to make plans for the future.	23.2	I would not want the the stress of knowing before symptoms start.	18.2
If he had DMD, I wanted to mentally prepare myself.	19.6	Other*: Risk for false positive.	18.2
Other*: To benefit others/help with research	19.6	Right now there is no cure or proven treatement.	13.6
		Other*: Do not want another needle stick or additional blood taken.	13.6
*Answers written in by questionnaire respondents.			

Evaluating Consent

Questionnaire results indicated that parents were able to make an informed decision about DMD screening when receiving information in the pediatric clinic (**Table 3**). Although about 20% of parents did not feel they had much time to review the materials, over 95% felt they understood the written information at least well enough or better and about 70% felt the written information was helpful to them in making a decision about participating. Nearly 95% of participants also reported that whoever spoke with them about screening was at least somewhat helpful in explaining the DMD screening program and they felt they were given enough time to ask questions.

**Table 3. Questions and respondent answers used to evaluate parents’ perceived usefulness of the information they received and their knowledge of Duchenne muscular dystrophy screening:** 

Question - Information received	Answer	%
Were you given any written information about screening for DMD? (n = 138)	Yes No Don't know/Don't remember	94.9 0.7 4.3
Did you feel you had enough time to read the written information? (n = 130)	Yes No Don't Know/Don't remember	78.5 17.7 3.8
How well did you understand the written information about DMD screening? (n = 125)	Very well/Well/Well enough Not well/Not at all	96.8 3.2
How helpful was the written information when deciding to have your son screened? (n = 128)	Very/Somewhat helpful Neutral Not very/Not helpful at all	68.8 24.2 7.0
Did anyone talk to you about the written information for screening? (n = 138)	Yes No Don't Know/Don't remember	90.6 4.3 5.1
How helpful was this person in explaining screening for DMD? (n = 123)	Very/Somewhat helpful Neutral Not very/Not helpful at all	94.3 4.1 1.6
Did this person give you enough time to ask questions about DMD screening? (n = 124)	Yes No Don't know/Don't remember	92.7 1.6 5.6
**Question - Knowledge (n = 135)**	**Correct Answer**	**%**
DMD is:	The most common form of muscular dystrophy	63.0
DMD is a genetic condition that weakens the:	All of the above (arm and leg, heart, and breathing muscles)	40.7
DMD can happen:	All of the above (in families with or without a relative with DMD, in all racial backgrounds)	60.7
Screening finds affected infants before symptoms start:	True	92.8
Once a child is found to have DMD:	Treatment may slow down the progress of the disorder	69.9

Over 95% of parents felt they understood the study information well and their answers to the knowledge questions generally reflected this (**Table 3**). The range of correct responses from a participant was 0 to 5, with a mean of 3.27 (SD. = 1.4). About three-quarters (74.6%) of respondents answered at least three of the five questions correctly. To determine whether the amount of time between screening and completion of the survey influenced the number of questions answered correctly, knowledge question results were compared between parents who answered the questionnaire from the first mailing and those who answered from the second mailing. The mean number of questions answered correctly between these two groups differed statistically (*P* < 0.01), with those answering the questionnaire from the first mailing (n = 60; mean= 3.57; SD = 1.2) answering more questions correctly than those who answered them at a later date (n = 61; mean = 2.90; SD = 1.4). Overall, these data showed that most parents had a good understanding of the information and also suggested that parents likely had an even better understanding of the risks and benefits of DMD screening at the time they made the decision to screen.

Parental Experience and Opinions

A large majority of respondents whose sons were screened reported a positive experience, with almost 90% of parents indicating that screening for DMD was worthwhile (**Table 4**). Another indication of a positive experience was that the majority of parents also reported that they would screen future sons and recommend screening to other parents.

Approximately 95% of parents reported that the screening experience was not stressful (**Table 4**). The low stress experienced by parents came as little surprise, as none of the parents received a positive screening result.

**Table 4. Parent, participant, and provider experiences and opinions of Duchenne muscular dystrophy screening:** 

Group	Question	Answer	%
Parents	Do you think boys should be screened for DMD? (n = 138)	Yes No Don't Know	65.2 0.0 34.8
	If yes, when should they be screened? (n = 89)	Right after birth Between 6 and 15 months of age When symptoms start Other	49.4 46.1 0.0 4.5
	Should screening be required or optional? (n = 138)	Required Optional No Opinion	12.3 78.3 9.4
	What describes your overall experience with DMD screening? (n = 137)	Very/Somewhat satified Neutral Very/Somewhat dissatisfied	73.7 16.1 9.5
	How stressful did you find your experience with DMD screening (n = 138)	Not at all/A little stressful Somewhat stressful Extremely/Very stressful	95.6 4.3 0.0
**Group**	**Statement**	% Disagreement*	**% Agreement† **
Parents	DMD screening was worthwhile. (n = 138)	0.0	87.0
	It is important for baby boys to be screened for DMD. (n = 135)	0.0	66.7
	Babies should be screened for as many disorders as possible even if it leads to false alarms. (n = 137)	24.9	44.6
	If I had another boy, I would want him to have DMD screening. (n = 137)	2.2	76.6
	I would recommend DMD screening to other parens (n = 137)	2.9	61.4
Providers	DMD screening can be easily incorporated into routine practive (n = 23)	0.0	100
	Collecting blood for the DMD screening test is not a burden (n = 21)	0.0	100
	Obtaining informed consent for DMD screening is not a burden. (n = 22)	4.5	72.7
	I am comfortable discussing DMD screening with my patients. (n = 19)	0.0	63.2
	I have the necessary information to discuss DMD screening with my patients. (n = 19)	5.3	79.0
	I am in favor of screening for DMD. (n = 22)	0.0	91.0
	I am satisfied with the information provided to educate parents about DMD screening. (n = 20)	0.0	95.0
	The benefits of DMD screening are greater than the risks. (n = 22)	0.0	90.9
*% Disagreement reflects the percentage of respondents who indicated that they disagreed or strongly disagreed with the statement.			
†% Agreement reflects the percentage of respondents who indicated that they agreed or strongly agreed with the statement.			

The majority of parents responding to the questionnaire were supportive of DMD screening (**Table 4**). Parents were interested in the option of DMD screening, particularly when performed along with routine testing. Written comments of parents indicated that they liked the convenience of including DMD screening with other tests, particularly as no additional needle stick was needed. Most parents thought screening should be optional, but they were evenly divided on the timing of screening, with half preferring it to be part of newborn screening and the other half preferring it to be done with other tests later in infancy. Although a majority of parents thought that identifying infants through screening was important, one-third did not think that screening for more conditions was indicated if it also increased the number of parents receiving false-positive results.

Provider Demographics and Opinions

A questionnaire was given to providers to evaluate their opinions about the DMD screening program. A total of 25 providers from three of the four clinics returned surveys. Most of those completing the provider questionnaire were female and White (**Table 1**). The majority of providers were nurses or physicians. Nurse practitioners, physician assistants, medical assistants, and laboratory technicians accounted for the rest of providers. All providers specialized in pediatrics and were in private practice in the metropolitan Atlanta area.

Information gathered through the provider questionnaire indicated that the majority of these providers were supportive of DMD screening (**Table 4**). Providers thought DMD screening could be incorporated easily into practice and that collecting blood, a role that the office staff routinely performed, was not a burden. The majority of providers also thought it was not a burden obtaining consent; however, as they were not involved in gaining parental consent, this could not be verified through practice.

## Discussion

This is the first study in the United States to conduct DMD screening after the newborn period through pediatric care providers. In Germany, where screening occurred through pediatric providers, parents were given information in the birthing hospitals and at pediatricians’ offices8, 9. Parents were then able to request screening during one of the well-baby visits from 4 weeks to 1 year of age. Scheuerbrandt et al reported that 14.4% of infant boys born in West Germany in 1983 were screened for DMD, with 65% of screening occurring at the first well-baby check at 4-6 weeks. At that time, they estimated that half the birthing hospitals in West Germany were providing information on screening.

Data from our study and from Germany demonstrate the benefits and limitations of infant screening in comparison with newborn screening for DMD. Screening after the newborn period enables some of the ethical issues surrounding newborn screening for DMD to be addressed. Parents could have more time to consider screening in a less hectic environment; our study showed that most parents who chose screening had enough time and were able to understand the risks and benefits of DMD screening. Later time points for screening also provide additional time for parent–child bonding to occur and clearly delineate between optional and mandatory screening. Because CK levels have been shown to be higher in the newborn period^31^, screening later in infancy when CK levels have normalized can decrease the rate of false positives; in our study, we did not detect any false positives while Scheuerbrandt et al had a low rate of 0.016%^8^. However, infant screening has several drawbacks when compared with newborn screening for DMD. While infant screening can occur at routine well-baby visits, established systems and policies are not in place for infant screening to be offered universally. Also, the later timing of infant screening means that some parents will not learn about their reproductive risks before having another child. Lastly, parents themselves might prefer screening to occur earlier; in our pilot study, half of the parents who participated preferred newborn screening to screening at a later age. Further analysis of parent preferences and experiences is planned through a direct comparison of questionnaire data from the CDC-funded newborn and infant screening studies.

The consideration of DMD newborn or later infant screening represents a recent shift in thinking about benefits not only to the affected child, but also to the family and society32, 33. Traditionally, benefits have focused on early medical interventions that improve the health outcome of affected children^17^. However, in 2005, along with proposing updated criteria and a set of 29 core conditions for newborn screening, the American College of Medical Genetics recommended that benefits to the family, providers, and public also should be considered after the best interest of the child^34^. Recently, the Secretary’s Advisory Committee on Heritable Disorders in Newborns and Children, the committee that reviews and recommends conditions for inclusion on newborn screening panels, published evaluation criteria that also consider benefits beyond those of the child^35^. Grosse et al describe this new paradigm shift as a change from viewing newborn screening as an urgent and immediate public health need (a public health emergency) to a public health service^33^. While DMD does not fulfill criteria for newborn screening under the old paradigm of a public health emergency, it could be considered for screening if done as a public health service. In that case, other childhood onset conditions, such as fragile X syndrome (FXS), that also are characterized by developmental delay in early childhood, a “diagnostic odyssey”, and a lack of therapies that can reverse or prevent disease also could be considered. Our results indicated that the majority of parents in our study were interested in DMD screening and likely would have considered screening for other childhood-onset conditions as well. Nevertheless, the timing of screening for these conditions might need to be different from those conditions in which screening clearly is a public health emergency. Although the public health system already is in place for early identification and follow-up of infants affected by DMD, screening for DMD later in infancy could avoid confusion of mingling mandatory and optional screening at birth and could reduce consent and other ethical concerns^23^. Infant screening for DMD and other childhood-onset conditions could be offered in pediatric offices as an optional public health service outside of newborn screening.

This study had several limitations. Although providers broadly supported screening, their questionnaire results must be interpreted with caution because clinic staff was not involved in the more time-consuming aspect of obtaining consent from parents. Having had clinic staff involved in educating potential participants would have enabled all parents of eligible infants to be educated about DMD screening and would have better reflected how screening would be carried out in routine practice. The study population was primarily White and highly educated. While clinics where recruitment occurred served diverse patient populations, those with larger minority populations had a higher number of patients who did not keep, rescheduled, or cancelled their appointments, making recruitment more difficult at these locations. Only about half of the participants completed the post-screening questionnaire and only about 20 decliners and 20 providers completed their questionnaires. The relatively small sample sizes for each type of questionnaire made it difficult to make meaningful comparisons between respondents and further reduced our ability to generalize the results to other populations. Since all infants had normal CK results, we were unable to evaluate the experiences of parents whose sons screened positive and the laboratory and clinical protocols for follow-up of infants with elevated CK.

Future studies will need to address several issues before DMD screening by pediatric providers can be incorporated into routine practice. Investigators conducting future infant screening studies should utilize clinic staff in conducting recruitment and obtaining parental consent, recruit a larger number of participants to determine the experience and test the follow-up of families with positive DMD results, evaluate the cost of screening, and ensure the participation of clinics (pediatric, family practice, and community clinics) that serve diverse pediatric populations. Having both the support and involvement of the clinic staff is essential. Kemper and Bailey recently surveyed pediatricians on their knowledge and attitudes toward FXS screening by posing scenarios of screening at birth through traditional newborn screening or at 12 months of age through pediatric providers^36^. FXS is similar to DMD in that both are genetic conditions for which (1) affected children are brought to medical attention through delays in development, (2) supportive therapies exist but there is no cure, and (3) diagnostic delay is common. Their results showed that 55% of respondents were supportive of FXS screening when infants were 12 months of age; however, 34% thought routine visits were too busy to include screening and only 39% felt comfortable discussing FXS with patients. While our study showed a higher proportion (63%) of clinical staff felt comfortable discussing DMD, both studies highlighted the need for provider education if they are to be involved in the screening process. In future studies, investigators will need to educate pediatric health care providers on DMD screening and provide them with informational resources for their patients. It also will be important to consult with providers about efficient ways to include screening that minimize the effects on routine practice.

This is the first study in the United States to conduct DMD screening after the newborn period. Parents and providers indicated broad support of voluntary DMD screening. Parents understood the risks and benefits of screening so that they could make informed choices. Our study indicated that DMD screening is feasible in a pediatric office. However, if infant screening for DMD and other childhood-onset conditions eventually is to be offered in pediatric provider offices as an optional public health service outside of newborn screening, future studies will need to assess the feasibility of DMD screening in routine practice by having providers play a larger role in educating and obtaining consent from parents. It is evident that larger studies and more public discussion need to occur before routine DMD infant screening can become a reality.

## Acknowledgments

We acknowledge several colleagues who were instrumental in conducting this study: Dr. Chunli Yu, Dr. Brad Coffee, Leonard Arthur, Barbara Adam, and Elizabeth McCown for developing, validating, and performing the laboratory testing; Hollie Lawyer and Aneidra Leysath for their role in educating and obtaining consent from parents; and Dr. Katherine Kolor for early involvement in planning the study. We also acknowledge advisory board members for their guidance and oversight of the study. Finally, we thank the staff of the four participating pediatric clinics, and the parents and their sons who participated in the study.

We would like to acknowledge our friend and colleague, Dr. Paul Fernhoff, who recently passed away. He worked tirelessly to improve the lives of children and families affected by genetic conditions and he will be greatly missed.

## Abbreviations List

DMD - Duchenne muscular dystrophy

CK - Creatine kinase

IRB - Institutional review board

CDC - Centers for Disease Control and Prevention

FXS - Fragile X syndrome

## References

[1] Bradley D, Parsons E. Newborn screening for Duchenne muscular dystrophy. Semin Neonatal. 1998;3:27-34.

[2] Drousiotou A, Ioannou P, Georgiou T, et al. Neonatal screening for Duchenne muscular dystrophy: a novel semiquantitative application of the bioluminescence test for creatine kinase in a pilot national program in Cyprus. Genet Test. 1998;2(1):55-60. 10.1089/gte.1998.2.55 10464597

[3] Emery AE. Duchenne Muscular Dystrophy. 2nd ed. New York: Oxford University Press; 1987.

[4] Ciafaloni E, Fox DJ, Pandya S, et al. Delayed diagnosis in Duchenne muscular dystrophy: data from the Muscular Dystrophy Surveillance, Tracking, and Research Network (MD STARnet). J Pediatr. Sep 2009;155:380-385. 10.1016/j.jpeds.2009.02.007 PMC5884059 19394035

[5] Crisp DE, Ziter FA, Bray PF. Diagnostic delay in Duchenne's muscular dystrophy. JAMA. Jan 22-29 1982;247:478-480. 7054550

[6] Mohamed K, Appleton R, Nicolaides P. Delayed diagnosis of Duchenne muscular dystrophy. Eur J Paediatr Neurol. 2000;4:219-223. 10.1053/ejpn.2000.0309 11030068

[7] Marshall PD, Galasko CS. No improvement in delay in diagnosis of Duchenne muscular dystrophy [letter]. Lancet. Vol 345. 1995/03/04 ed1995:590-591. 10.1016/s0140-6736(95)90503-0 7605512

[8] Scheuerbrandt G, Lundin A, Lovgren T, Mortier W. Screening for Duchenne muscular dystrophy: an improved screening test for creatine kinase and its application in an infant screening program. Muscle Nerve. Jan 1986;9:11-23. 10.1002/mus.880090103 3951477

[9] van Ommen GJ, Scheuerbrandt G. Neonatal screening for muscular dystrophy. Consensus recommendation of the 14th workshop sponsored by the European Neuromuscular Center (ENMC). Neuromuscul Disord. May 1993;3:231-239. 10.1016/0960-8966(93)90065-r 8400865

[10] Zellweger H, Antonik A. Newborn screening for Duchenne muscular dystrophy. Pediatrics. Jan 1975;55:30-34. 1110862

[11] Drummond LM. Creatine phosphokinase levels in the newborn and their use in screening for Duchenne muscular dystrophy. Arch Dis Child. May 1979;54(5):362-366. 10.1136/adc.54.5.362 PMC1545573 475411

[12] Plauchu H, Cordier MP, Carrier HN, et al. Systematic neonatal detection of Duchenne's muscular dystrophy. Results after 10 years' of experience in Lyons (France). J Genet Hum. Aug 1987;35(4):217-230. 3655748

[13] Skinner R, Emery AE, Scheuerbrandt G, Syme J. Feasibility of neonatal screening for Duchenne muscular dystrophy. J Med Genet. Feb 1982;19(1):1-3. 10.1136/jmg.19.1.1 PMC1048810 7069739

[14] Eyskens F, Philips E. Newborn screening for Duchenne muscular dystrophy. The experience in the province of Antwerp. Neuromuscular Disorders. 2006;16:721.

[15] Greenberg CR, Jacobs HK, Halliday W, Wrogemann K. Three years' experience with neonatal screening for Duchenne/Becker muscular dystrophy: gene analysis, gene expression, and phenotype prediction. Am J Med Genet. Apr 1 1991;39(1):68-75. 10.1002/ajmg.1320390115 1867267

[16] Naylor E, Hoffman E, Paulus-Thomas J, et al. Neonatal screening for Duchenne/Becker muscular dystrophy: reconsideration based on molecular diagnosis and potential therapeutics. Screening. 1992;1:99-113.

[17] Wilson JM, Jungner YG. Principles and practice of screening for disease. Public Health Papers. no. 34. Geneva: World Health Organization. 1968:163p.

[18] Bushby K, Finkel R, Birnkrant DJ, et al. Diagnosis and management of Duchenne muscular dystrophy, part 1: diagnosis, and pharmacological and psychosocial management. Lancet Neurol. Jan 2010;9:77-93. 10.1016/S1474-4422(09)70271-6 19945913

[19] Bushby K, Finkel R, Birnkrant DJ, et al. Diagnosis and management of Duchenne muscular dystrophy, part 2: implementation of multidisciplinary care. Lancet Neurol. Feb 2010;9:177-189. 10.1016/S1474-4422(09)70272-8 19945914

[20] Moxley RT, Ashwal S, Pandya S, et al. Practice parameter: corticosteroid treatment of Duchenne dystrophy: report of the Quality Standards Subcommittee of the American Academy of Neurology and the Practice Committee of the Child Neurology Society. Neurology. Jan 11 2005;64:13-20. 10.1212/01.WNL.0000148485.00049.B7 15642897

[21] American Academy of Pediatrics Section on Cardiology and Cardiac Surgery. Cardiovascular health supervision for individuals affected by Duchenne or Becker muscular dystrophy. Pediatrics. Dec 2005;116:1569-1573. 10.1542/peds.2005-2448 16322188

[22] Finder JD, Birnkrant D, Carl J, et al. Respiratory care of the patient with Duchenne muscular dystrophy: ATS consensus statement. Am J Respir Crit Care Med. Aug 15 2004;170(4):456-465. 10.1164/rccm.200307-885ST 15302625

[23] Ross LF. Screening for conditions that do not meet the Wilson and Jungner criteria: the case of Duchenne muscular dystrophy. Am J Med Genet A. Apr 15 2006;140:914-922. 10.1002/ajmg.a.31165 16528755

[24] American Society of Human Genetics (ASHG) American College of Medical Genetics (ACMG). Points to consider: ethical, legal, and psychosocial implications of genetic testing in children and adolescents. Am J Hum Genet. Nov 1995;57:1233-1241. PMC1801355 7485175

[25] Hewlett J, Waisbren SE. A review of the psychosocial effects of false-positive results on parents and current communication practices in newborn screening. J Inherit Metab Dis. Oct 2006;29:677-682. 10.1007/s10545-006-0381-1 16917730

[26] Fuentes-Afflick E, Hessol NA. Interpregnancy interval and the risk of premature infants. Obstet Gynecol. Mar 2000;95:383-390. 10.1016/s0029-7844(99)00583-9 10711549

[27] Mendell JR, Shilling C, Leslie ND, et al. Evidence-based path to newborn screening for duchenne muscular dystrophy. Annals of neurology. Mar 2012;71(3):304-313. 10.1002/ana.23528 22451200

[28] Orfanos AP, Naylor EW. A rapid screening test for Duchenne muscular dystrophy using dried blood specimens. Clin Chim Acta. Apr 27 1984;138:267-274. 10.1016/0009-8981(84)90133-5 6723063

[29] American Academy of Pediatrics Committee on Nutrition. Iron fortification of infant formulas. Pediatrics. Jul 1999;104:119-123. 10390274

[30] Statistical Package for the Social Sciences (SPSS) [computer program]. Version 15.0. Chicago, IL: SPSS, Inc.; 2006.

[31] Blum D, Brauman J. Serum enzymes in the neonatal period. Diagnostic aid in muscle pathology. Biol Neonate. 1975;26(1-2):53-57. 10.1159/000240716 1148340

[32] Bailey DB, Jr., Beskow LM, Davis AM, Skinner D. Changing perspectives on the benefits of newborn screening. Ment Retard Dev Disabil Res Rev. 2006;12:270-279. 10.1002/mrdd.20119 17183569

[33] Grosse SD, Boyle CA, Kenneson A, Khoury MJ, Wilfond BS. From public health emergency to public health service: the implications of evolving criteria for newborn screening panels. Pediatrics. Mar 2006;117:923-929. 10.1542/peds.2005-0553 16510675

[34] American College of Medical Genetics Newborn Screening Expert Group. Newborn screening: toward a uniform screening panel and system--executive summary. Pediatrics. May 2006;117:S296-307. 10.1542/peds.2005-2633I 16735256

[35] Calonge N, Green NS, Rinaldo P, et al. Committee report: Method for evaluating conditions nominated for population-based screening of newborns and children. Genet Med. Mar 2010;12(3):153-159. 10.1097/GIM.0b013e3181d2af04 20154628

[36] Kemper AR, Bailey DB, Jr. Pediatricians' knowledge of and attitudes toward fragile X syndrome screening. Acad Pediatr. Mar-Apr 2009;9:114-117. 10.1016/j.acap.2008.11.011 19329102

